# Study on Synergistically Improving Corrosion Resistance of Microarc Oxidation Coating on Magnesium Alloy by Loading of Sodium Tungstate and Silane Treatment

**DOI:** 10.3390/ma18020361

**Published:** 2025-01-14

**Authors:** Ziyi Wang, Lingyun An, Chenggong Chang, Leichao Meng, Donghao Lei, Jianhong Peng, Zhanying Wang

**Affiliations:** 1 Qinghai Provincial Key Laboratory of Nanomaterials and Technology, School of Chemistry and Materials Science, Qinghai Minzu University, Xining 810007, China; zy2534929264@126.com (Z.W.); leichao5166@163.com (L.M.); 18822203344@163.com (D.L.); pjhhj@sohu.com (J.P.); 15193195979@163.com (Z.W.); 2 Key Laboratory of Comprehensive and Highly Efficient Utilization of Salt Lake Resources, Qinghai Institute of Salt Lake, Chinese Academy of Sciences, Xining 810008, China; 3 Key Laboratory of Salt Lake Resources Chemistry of Qinghai Province, Xining 810008, China

**Keywords:** magnesium alloy, microarc oxidation, sodium tungstate, silane, microstructure, corrosion resistance

## Abstract

Sodium tungstate (Na_2_WO_4_) was filled into the micropores and onto the surface of a magnesium alloy microarc oxidation (MAO) coating by means of vacuum impregnation. Subsequently, the coating was sealed through silane treatment to synergistically boost its corrosion resistance. The phase composition of the coating was inspected using XRD. FTIR was utilized to analyze the functional groups in the coating. XPS was employed to study the chemical composition and valence state of the coating. The surface and cross-sectional morphology of the coating, along with its elemental composition and distribution, were investigated by SEM and EDS. Meanwhile, the thickness of the coating was analyzed using Image J software. Electrochemical impedance spectroscopy (EIS) was employed to determine the corrosion resistance of the coating. The results show that compared with an MAO coating, M-0.125W composite coating (only filled with sodium tungstate on the surface of the MAO coating), and M-SG composite coating (only receiving silanization treatment applied to the surface of the MAO coating), the corrosion resistance of the M-nW-SG composite coating (loaded with sodium tungstate on the surface of the MAO coating and then treated with silane) is significantly improved. This is mainly attributed to the fact that sodium tungstate can be combined with Mg^2+^ to form insoluble magnesium tungstate protective film, which blocks corrosion media. At the same time, silanization treatment further seals the MAO coating and increases the compactness of the coating. In addition, with the increase in the impregnation concentration of sodium tungstate, the content of sodium tungstate in the M-nW-SG composite coating improves, and the sealing effect of silanization treatment is better. When the impregnation concentration of sodium tungstate is 0.1 mol/L or above, the MAO coating with sodium tungstate can be completely sealed. When the impregnation concentration of sodium tungstate is 0.125 mol/L, M-0.125W-SG composite coating has the best corrosion resistance, and its impedance modulus value can be maintained at 8.06 × 10^6^ Ω·cm^2^ after soaking in 3.5 wt.% NaCl solution for 144 h, which is about three orders of magnitude higher than those of MAO coating and M-0.125W and M-SG composite coatings.

## 1. Introduction

Magnesium alloy is extensively utilized in fields like automotive and aerospace because of its lightweight, high-specific-strength, favorable damping, and outstanding electromagnetic shielding properties [1,2]. Nonetheless, the standard electrode potential of magnesium alloy is negative, standing at merely −2.37 V, which leads to its tendency to corrode easily. Consequently, enhancing the corrosion resistance of magnesium alloys is an important issue that needs to be solved urgently for their further development and application [3,4,5]. The methods for enhancing the corrosion resistance of magnesium alloys include alloying treatment, optimization of the preparation process, surface modification. and so on. Among these, surface modification technology is favored by researchers because it is environmentally friendly, is highly adjustable, and has a good protective effect. At present, a series of surface modification technologies have been developed to improve the corrosion resistance of magnesium alloys, such as electroless plating [6], organic coating [7], vapor deposition [8,9], anodic oxidation [10], and microarc oxidation [11,12,13].

Microarc oxidation (MAO), also known as plasma electrolytic oxidation (PEO), is a ceramic coating that is dominated by matrix metal oxides and is in situ grown on the surface of a metal substrate under high voltage [14,15]. MAO coating has the advantages of high corrosion resistance, excellent wear resistance, and strong bonding with the metal substrate [16,17], and thus, it has a broad application prospect. Generally, MAO coating consists of an inner dense layer and an outer loose, porous layer. However, the existence of the loose, porous layer reduces its corrosion resistance to magnesium alloys [18]. Therefore, various methods have been put forward to improve the microdefects of magnesium alloy MAO coating, such as optimizing the process parameters of microarc oxidation, sol-gel sealing [19], and the in situ growth of layered double hydroxides (LDHs) [20], among others. Nevertheless, these methods can only play the role of passive physical barriers, and there is still a certain disparity from the ideal long-term protection. Hence, the use of corrosion inhibitors has emerged.

A corrosion inhibitor is a chemical substance that can effectively slow down or prevent the corrosion of metal materials by means of physical or chemical adsorption on the metal matrix when the corrosive medium is close to or in contact with the metal to be protected [21]. The commonly used method for corrosion inhibitors is direct doping into the coating. However, this destroys the ordered structure of the coating, reduces the binding force between the coating and the substrate, and even causes the coating to fall off [22,23]. Therefore, in recent years, many scholars have focused their research on “smart” micro–nanocontainers. They load corrosion inhibitors on the surface or inside micro–nanocontainers and then dope them into the coating to avoid affecting the overall integrity of the coating and achieve controlled release of corrosion inhibitors [24]. For instance, Kordas et al. [25] prepared an epoxy resin composite coating by incorporating TiO_2_ nanocontainers loaded with 8-hydroxyquinoline (8-HQ), which demonstrated enhanced corrosion resistance. Liu et al. [26] synthesized a novel graphene/β-cyclodextrin-based supramolecular nanocapsule. Subsequently, they used the nanocapsule loaded with benzotriazole (BTA) to endow the coating with excellent passive and active anticorrosion properties. The results show that the long-term corrosion resistance of the coating is remarkably improved. However, the preparation process of a “smart” micro–nanocontainer is often complicated, and when it is uniformly distributed in the coating, due to the interaction of chemical bonds, the corrosion inhibitor is difficult to diffuse to interface between the coating and metal substrate in time to play an effective protective role [27]. Moreover, the corrosion inhibitor is exposed to the environment outside of the coating and can easily diffuse into the corrosion medium, resulting in the waste of the corrosion inhibitor and a reduction in its protective effect.

In recent years, some scholars have made attempts to utilize the micropores of MAO coating as containers for loading corrosion inhibitors. For instance, Akbarzadeh et al. [28] successfully prepared an MAO coating with self-healing properties on AA2024 aluminum alloy by taking advantage of defects such as micropores in the MAO coating as a repository for corrosion inhibitors. Lamaka et al. [29] loaded corrosion inhibitors into the pores of an MAO coating and then sealed them with sol-gel hybrid polymers. The results indicated that the thickness of the prepared composite coating doped with Ce^3+^ ions was approximately 3.7 to 7.0 μm, and it provided effective corrosion protection for the magnesium alloy. Therefore, it can be observed that using the microdefects of an MAO coating as a container for loading corrosion inhibitors is feasible. This approach can not only avoid the problem of the coating’s integrity being affected by directly doping the corrosion inhibitor into the coating but also make full use of the microdefects in the MAO coating.

A large number of studies have proved that sodium tungstate is an effective and environmentally friendly anodic corrosion inhibitor in the steel field, which can form iron–tungstate complexes on the surface of steel to protect steel from corrosion in neutral, acidic, and alkaline solutions [30]. Liu et al. [31] embedded sodium tungstate in talc powder and subsequently added it to an epoxy resin coating to obtain a self-healing coating on the surface of tin-plated steel plate. The results showed that the impedance of the coating remained above 10^9^ Ω·cm^2^ after soaking in 3.5 wt.% NaCl solution for 25 days. Gaber et al. [32] investigated the corrosion behavior of iron-based alloys in sulfuric acid solutions of different concentrations ranging from 1% to 10%. They pointed out that sodium tungstate possessed a good corrosion inhibition effect on iron-based alloys in a 10% H_2_SO_4_ solution. And when the concentration of sodium tungstate was 0.1 mol /L, the corrosion density was the lowest. Additionally, Tu et al. [33] also added sodium tungstate to an aluminate-base electrolyte and prepared a W-containing ceramic coating on an AZ31 magnesium alloy surface by the MAO technique. The results showed that the addition of sodium tungstate to the electrolyte significantly improved the corrosion resistance of the MAO coating.

In this paper, different concentrations of sodium tungstate were loaded into the micropores and on the surface of an MAO coating through a vacuum impregnation method. Subsequently, the coating was sealed by silane treatment using a silane solution with 3-glycylglyoxypropyl trimethoxy silane (GPTMS) as the precursor. The effects of the loaded content of sodium tungstate and silane treatment on the microstructure and corrosion resistance of the composite coating were investigated, and the influence mechanism was explored.

## 2. Experiment and Method

### 2.1. Substrate Pretreatment

In this experiment, AZ31B magnesium alloy discs with a diameter of 45 mm and a thickness of 3 mm were chosen as the substrate and were supplied from Jiasheng Chemical Co., Ltd. (Lanzhou, China). The chemical compositions of these discs are presented in Table 1. The magnesium alloy substrate was successively polished with 400#, 800#, 1200#, and 2000# SiC sandpaper to remove impurity particles, contaminants, and the oxide layer on its surface, which was beneficial to the uniform growth of the subsequent MAO coating. Subsequently, ultrasonic cleaning was performed with alcohol for 10 min. After that, it was dried with a hair dryer and bagged for later use.

### 2.2. Preparation of MAO Coating

AZ31B magnesium alloy substrate was treated for 10 min by using an RZWH20A-II bipolar pulse microarc oxidation power supply (Rizhao microarc technology Co., Ltd., Rizhao, China) in an electrolyte solution containing Na_2_SiO_3_·9H_2_O (15 g/L), NaOH (8 g/L), and KF (8 g/L) under the conditions of 350 V voltage, 1000 Hz pulse frequency, and 12% duty ratio. During the process of preparing the MAO coating, the magnetic stirrer was continuously stirred to ensure that the MAO electrolyte was in a flowing state so as to ensure the uniformity of the electrolyte during preparation of the coating. The cooling device was used to control the temperature of the electrolyte at about 20 °C to prevent the failure of the electrolyte due to high temperature.

### 2.3. Loading of Sodium Tungstate

Using deionized water as the solvent, sodium tungstate aqueous solutions with concentrations of 0.025 mol/L, 0.05 mol/L, 0.1 mol/L, and 0.125 mol/L were, respectively, prepared. The pH of these solutions was adjusted to 11 by using a 2 mol/L NaOH solution. Then, the MAO coating was immersed in the prepared sodium tungstate aqueous solution with different concentrations, impregnated in a vacuum-drying oven (DZF, Lichen Instrument Technology Co., Ltd., Shanghai, China) for 6 h, and dried by cold air with a hair dryer to release the load of sodium tungstate into micropores and on the surface of the MAO coating.

### 2.4. Preparation of Silane Film

Anhydrous ethanol, 3-glycidyloxypropyltrimethoxysilane (GPTMS), and deionized water were mixed at a ratio of 43.3:33.7:21, stirring at room temperature for 1 h to produce GPTMS solution with a pH of 5.5.

**Figure 1:**
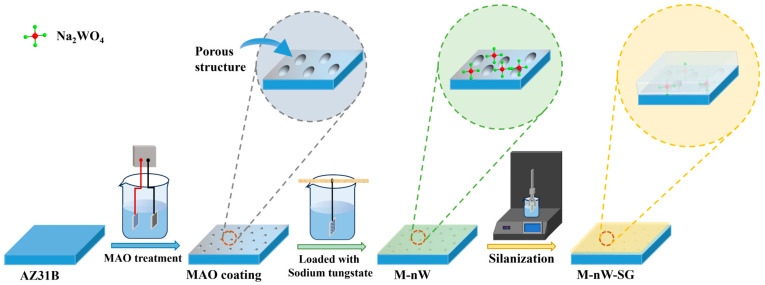
Schematic diagram of preparation process of M-nW-SG composite coating.

**Figure 2:**
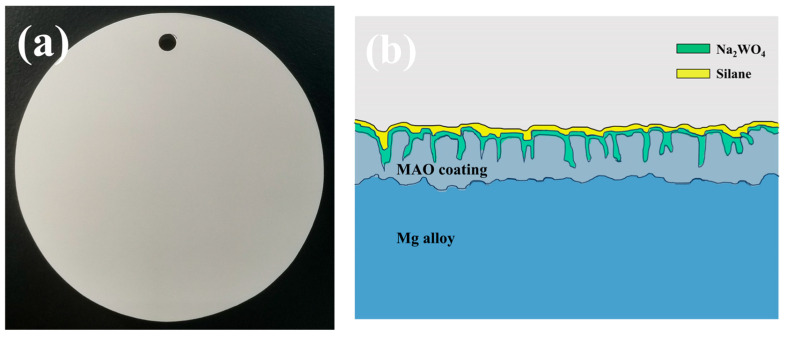
Actual macroscopical image (**a**) and diagrammatic illustration of cross-sectional structure (**b**) of M-nW-SG composite coating.

The sodium tungstate-loaded MAO coating was further silanized by using an SYDC-100 dipping machine (Three research technology Co., Ltd., Shanghai, China). The specific operation procedure is as follows: Immerse the MAO coating loaded with different concentrations of sodium tungstate into the GPTMS solution at a speed of 100 mm/min for 5 min. Then, take it out at the same speed and cure it at room temperature for 15 min. Repeat the above operation three times. Subsequently, transfer it to the oven and cure it at 60 °C for 1 h and at 100 °C for 24 h. Finally, take it out and cool it at room temperature. Thus, M-nW-SG composite coatings are prepared on the magnesium alloy. The schematic diagram of the preparation process of the M-nW-SG composite coating is shown in Figure 1, and its actual macroscopic image and cross-sectional structure are shown in Figure 2. The specific names of the prepared coatings are listed in Table 2. Among them, M-0.125W refers to only loading 0.125 mol/L sodium tungstate on the surface of the MAO coating. M-SG indicates that only silanization is performed on the surface of the MAO coating. M-0.025W-SG, M-0.05W-SG, M-0.1W-SG, and M-0.125W-SG, respectively, represent the MAO coatings on which sodium tungstate with concentrations of 0.025 mol/L, 0.05 mol/L, 0.1 mol/L, and 0.125 mol/L is loaded and then silanization is performed.

### 2.5. Characterization

A Thermo Fisher Scientific-Apreo 2C field emission scanning electron microscope (SEM) (Thermo Fisher Scientific Inc., Waltham, MA, USA) was used to study the surface and cross-section morphology of the coating. Meanwhile, the chemical composition and element distribution of the surface and cross section of the coating were measured twice by energy dispersive X-ray spectroscopy (EDS) to ensure the accuracy of the data. Image J software (version 1.52) was used to measure the thickness of the coating on the obtained cross-section image at least 10 times. An X-ray diffractometer (XRD, Rigaku Ultima IV, Tokyo, Japan) was used to detect the phase composition of the coating; the scanning range was 5~80°, the scanning rate was 10°/min, and the anode was a copper target. Fourier transform infrared spectroscopy (FT-IR, Great 10, Zhongke Ruijie Technology Co., Ltd., Tianjin, China) was used to analyze the functional groups of the coating, and the wave number range was 400 cm^−1^ to 4000 cm^−1^. The specific operation is as follows. First, the coating was peeled off the magnesium alloy matrix using a lancet, mixed with KBr and ground, and pressed into sheets for testing. At the same time, for comparative analysis, tiny droplets of silane solution were coated on the glass sheet, cured according to the steps mentioned in Section 2.4, pressed, and FT-IR tested, and the sample was named SG. An X-ray photoelectron spectrometer (XPS, Thermo Scientific-ESCALAB Xi+, Waltham, MA, USA) was used to study the chemical composition and valence state of the coating. Al-Kα was used as the excitation source, and its photoelectron energy was 1486.6 eV, which was calibrated at C 1s (284.8 eV). Avantage software (version 5.948) was used to fit the element fine spectrum by peaks.

### 2.6. Electrochemical Analysis

The corrosion resistance of MAO and its composite coatings was evaluated by electrochemical impedance spectroscopy (EIS) in 3.5 wt.% NaCl solution using a CHI660E electrochemical workstation (Chenhua Instrument Co., Ltd., Shanghai, China) with a standard three-electrode system. MAO and its composite coatings were used with an exposed area of 1 cm^2^ as the working electrode; the reference electrode was a saturated calomel electrode, and a platinum sheet was used as the opposite electrode. The initial potential was open circuit potential, the frequency range was 10^5^~10^−2^ Hz, the disturbance potential was 10 mV, and the tests were carried out according to the soaking time of 10, 24, 48, 72, 96, 120, and 144 h. For each coating, at least three electrochemical tests were performed to ensure repeatability of the results, and a fitting analysis of the electrochemical impedance spectrum was performed using ZSimpWin 3.2 software.

## 3. Results

### 3.1. XRD Analysis

**Figure 3:**
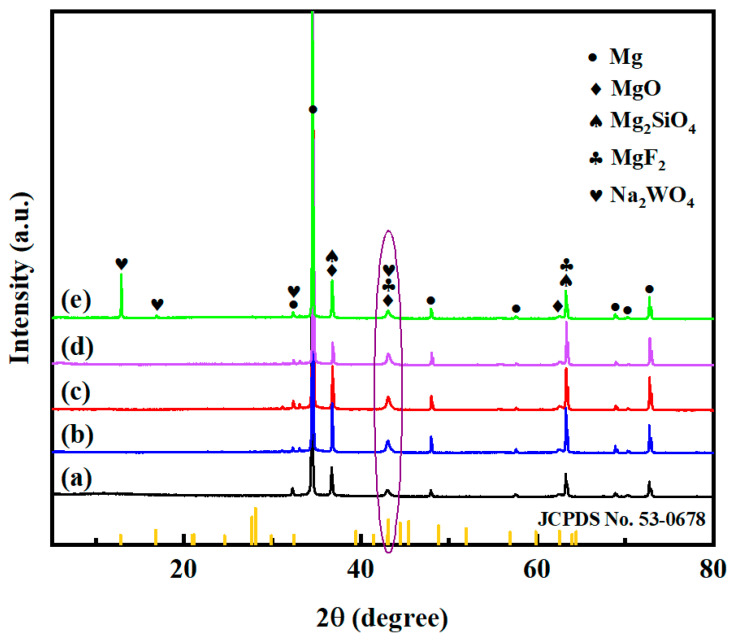
XRD patterns of MAO (a) and its composite coatings formed on AZ31B magnesium alloys: (b) M-0.025W-SG; (c) M-0.05W-SG; (d) M-0.1W-SG; (e) M-0.125W-SG.

The XRD patterns of MAO and its composite coatings are shown in Figure 3. As can be seen from Figure 3, both the MAO coating and its composite coatings are mainly composed of MgO (JCPDS No. 71-1176), Mg_2_SiO_4_ (JCPDS No. 87-2031), and MgF_2_ (JCPDS No. 70-0212) phases, while the stronger Mg diffraction peaks (JCPDS No. 89-4244) come from the matrix. This indicates that the magnesium alloy matrix and electrolyte ions are involved in the complex microarc oxidation process, and the possible formation reactions are shown in Equations (1)–(8).

In the process of microarc oxidation, the magnesium alloy substrate is firstly dissolved into Mg^2+^ ions according to Equation (1), which migrate outward along the discharge channel under high voltage and react with O^2−^, OH^−^, SiO_3_^2−^, and F^−^ ions from the electrolyte to form MgO, Mg_2_SiO_4_, and MgF_2_ (Equations (2)–(8)).

Mg → Mg^2+^ + 2e^−^ (1)

2H_2_O + 2e^−^ → H_2_ + 2OH^−^ (2)

2OH^−^ → O^2−^ + H_2_O (3)

Mg^2+^ + O^2−^ → MgO (4)

Mg^2+^ + 2OH^−^ → Mg(OH)_2_ (5)

Mg(OH)_2_ → MgO + H_2_O (6)

Mg^2+^ + 2F^−^ → MgF_2_ (7)

2Mg^2+^ + SiO_3_^2−^ + 2OH^−^ → Mg_2_SiO_4_ + H_2_O (8)

In addition, after sodium tungstate is loaded into the micropores and onto the surface of the magnesium alloy MAO coating, the diffraction peaks of sodium tungstate (JCPDS No. 53-0678) also appear; at the same time, the diffraction peak intensity at 2θ of 43.1° is enhanced, as shown by the purple oval in Figure 3, indicating that sodium tungstate has been successfully loaded into the micropores and on the surface of the MAO coating. However, with the increase in sodium tungstate concentration, it can be observed that there is no significant difference in the diffraction peak intensity. Since the amount of sodium tungstate added is small, it is difficult to notice any significant changes.

### 3.2. FT-IR Analysis

**Figure 4:**
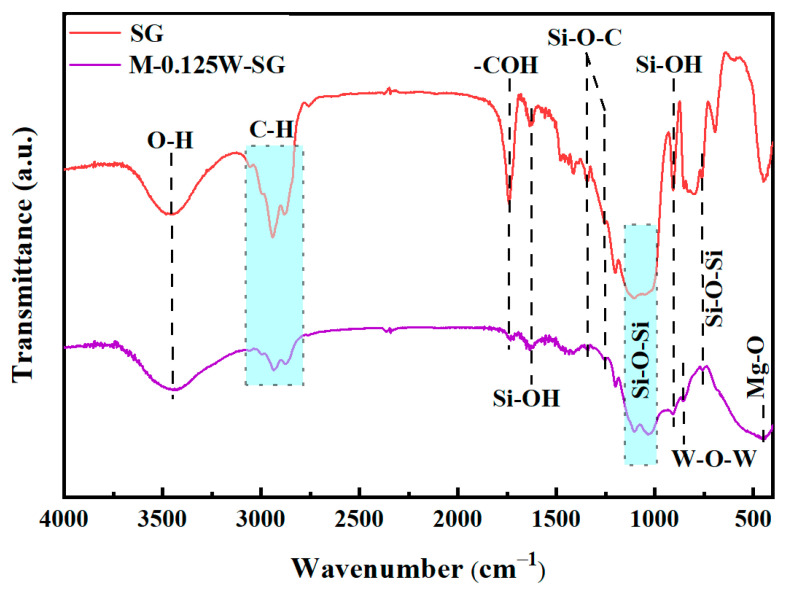
FT-IR spectra of SG sample and M-0.125W-SG composite coating.

Figure 4 shows the FT-IR spectra of the SG sample and M-0.125W-SG composite coating. As can be seen from Figure 4, the peak at 3455 cm^−1^ can be observed in both the SG sample and the M-0.125W-SG composite coating, which corresponds to the stretching vibration of the O-H bond of GPTMS [34]. The peaks at 3053 cm^−1^, 2989 cm^−1^, 2937 cm^−1^, and 2873 cm^−1^ are attributed to the stretching vibration of the C-H bond [35]. The peak at 1744 cm^−1^ corresponds to the stretching vibration of -COH [36]. The peaks at 1628 cm^−1^ and 902 cm^−1^ come from the bending vibration of O-H in Si-OH, which is related to the hydrolytic condensation reaction of silanization [37,38]. There are Si-O-C groups at 1345 cm^−1^ and 1253 cm^−1^ [39,40]. The peaks at 1101 cm^−1^ and 1034 cm^−1^ correspond to the Si-O-Si asymmetric stretching vibration. This indicates that there is silane film on the surface of the MAO coating on the magnesium alloy, which is formed by a condensation reaction of hydrolyzed GPTMS molecules on the surface of the coating. In addition, the M-0.125W-SG composite coating shows a new characteristic peak at 853 cm^−1^, which is attributed to the stretching vibration of W-O-W [41], demonstrating the successful loading of sodium tungstate on the surface of the MAO coating

### 3.3. XPS Analysis

**Figure 5:**
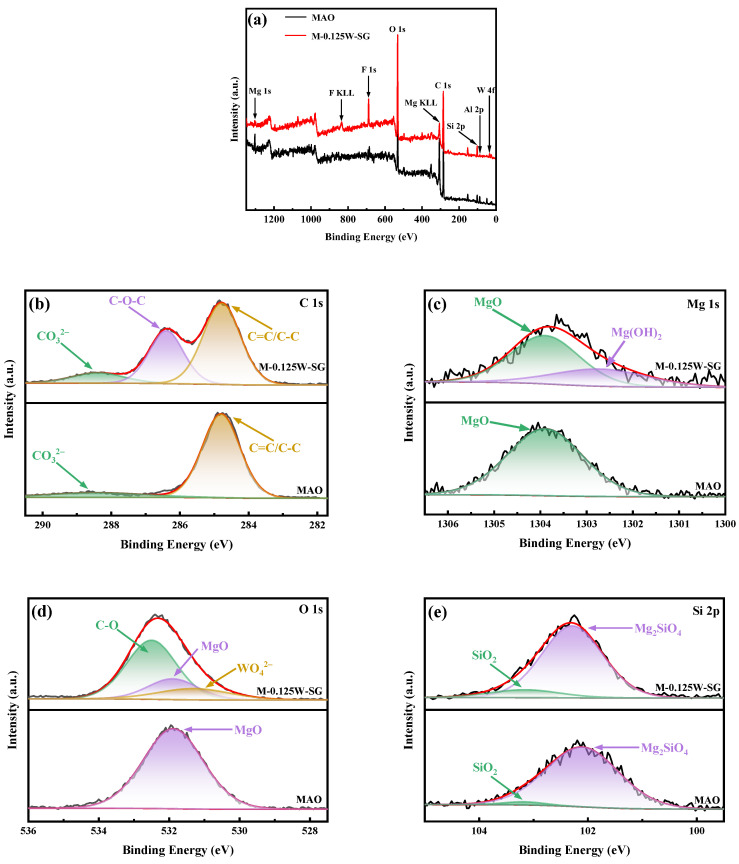

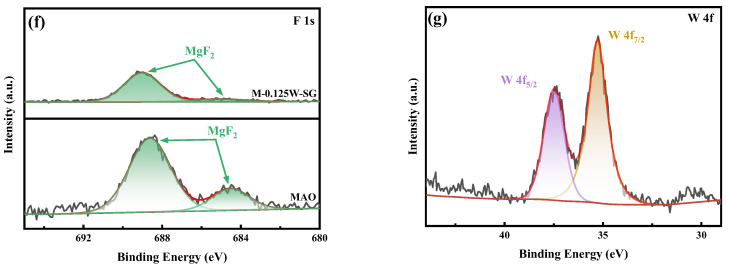
XPS spectra of MAO coating and M-0.125W-SG composite coating obtained on AZ31B magnesium alloys: (**a**) survey spectrum; high-resolution spectra of (**b**) C 1s, (**c**) Mg 1s, (**d**) O 1s, (**e**) Si 2p, (**f**) F 1s, and (**g**) W 4f.

Figure 5 shows the XPS survey spectra and XPS high-resolution spectra of the MAO coating and M-0.125W-SG composite coating. It can be seen from Figure 5a that both the MAO coating and M-0.125W-SG composite coating contain Mg, Al, O, Si, F, and C elements. Among them, Mg and Al are derived from the AZ31B magnesium alloy substrate, while O, Si, and F are derived from the electrolyte. In addition, the W element is also detected in the M-0.125W-SG composite coating.

To further analyze the valence state and existence form of elements in the coating, the high-resolution XPS spectra of the coating are fitted and analyzed. Figure 5b shows the fine spectrum of C 1s. Compared with the MAO coating, the C 1s in the M-0.125W-SG composite coating is divided into three fitting peaks, among which 284.8 eV and 288.4 eV represent foreign hydrocarbons, surface hydrocarbons, and CO_3_^2−^ [42], respectively, while the peak at 286.3 eV represents the C-O-C group. The results show that there is silane film on the surface of the M-0.125W-SG composite coating. In the Mg 1s XPS spectrum, Mg 1s in the M-0.125W-SG composite coating can fit two binding energy peaks at 1303.9 eV and 1302.8 eV (Figure 5c), which are attributed to MgO and Mg-OH bonds in Mg(OH)_2_ [43,44]. This suggests the formation of a layer of Mg(OH)_2_ precipitate on the surface of the MAO coating due to the influence of the pH value and aqueous solution environment during the vacuum impregnation of sodium tungstate. As can be seen from Figure 5d, the O 1s high-resolution spectra of the M-0.125W-SG composite coating can fit three peaks, among which the peak at 531.2 eV corresponds to the sodium tungstate supported on the surface of the MAO coating [45], and the peak at 531.9 eV corresponds to the main component MgO of the MAO coating. The higher peak at 532.5 eV corresponds to C-O, which is derived from the silane film on the surface of the MAO coating. These indicate that the surface of the MAO coating is successfully loaded with sodium tungstate and coated with silane film. In Figure 5e, in addition to Mg_2_SiO_4_ corresponding to 102.1 eV, SiO_2_ corresponding to 103.2 eV also appears, and it is higher in the M-0.125W-SG composite coating than in the MAO coating. This shows that more SiO_2_ in the M-0.125W-SG composite coating comes from the silane film. Figure 5f shows the F 1s spectrum, corresponding to MgF_2_ in the MAO coating. In Figure 5g, the characteristic peaks at 35.3 eV (W 4f_7/2_) and 37.5 eV (W 4f_5/2_) are attributed to W^6+^ bimodal [41], which proves that the M-0.125W-SG composite coating contains tungstate ions and further verifies that sodium tungstate is successfully loaded on the surface of the magnesium alloy MAO coating.

### 3.4. Surface Morphology and Elemental Composition and Distribution

**Figure 6:**
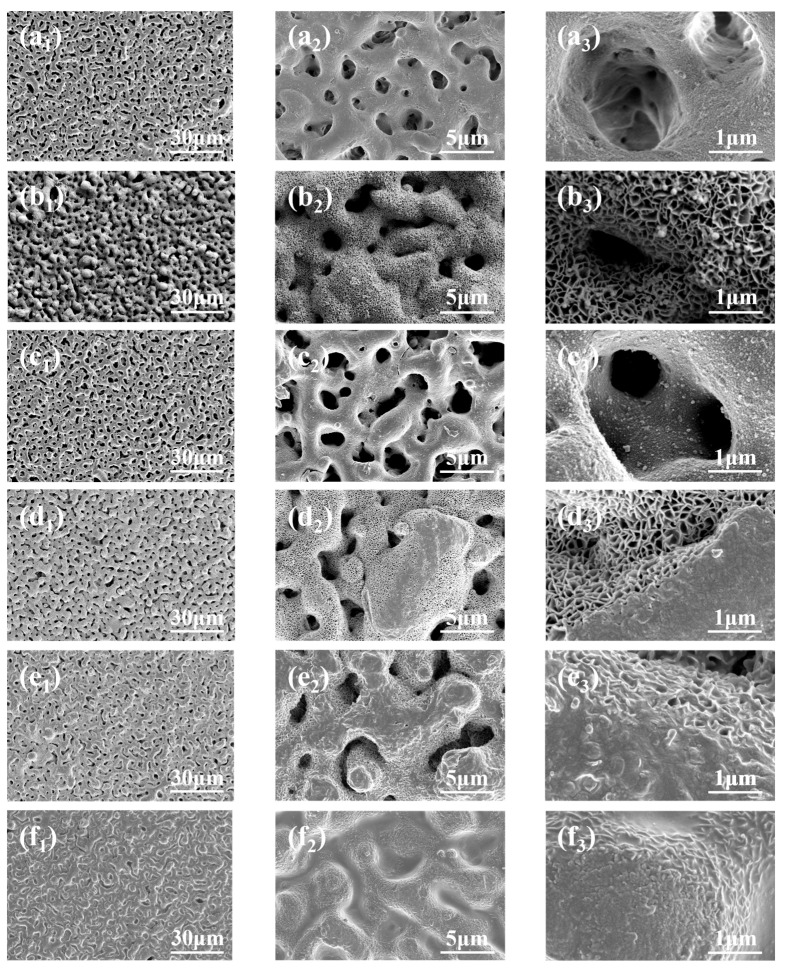

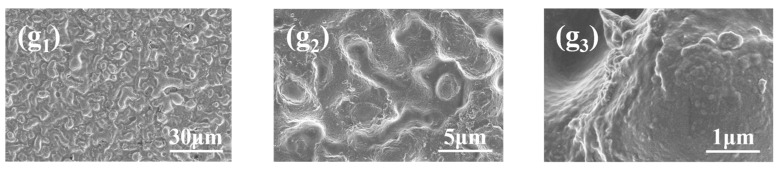
Surface morphologies of MAO (**a_1_**–**a_3_**) and its composite coatings formed on AZ31B magnesium alloys: (**b_1_**–**b_3_**) M-0.125W; (**c_1_**–**c_3_**) M-SG; (**d_1_**–**d_3_**) M-0.025W-SG; (**e_1_**–**e_3_**) M-0.05W-SG; (**f_1_**–**f_3_**) M-0.1W-SG; (**g_1_**–**g_3_**) M-0.125W-SG.

**Figure 7:**
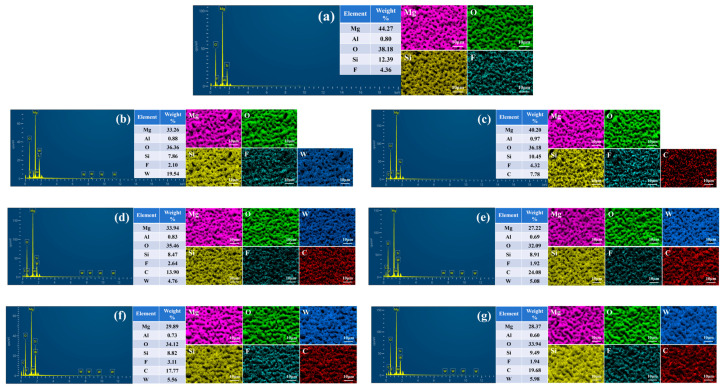
Composition and distribution of elements of MAO (**a**) and its composite coatings formed on AZ31B magnesium alloys: (**b**) M-0.125W; (**c**) M-SG; (**d**) M-0.025W-SG; (**e**) M-0.05W-SG; (**f**) M-0.1W-SG; (**g**) M-0.125W-SG.

Figure 6 and Figure 7 show the surface morphology, element composition, and distribution of different coatings, respectively. As can be seen from Figure 6a_1_,a_2_, the surface of the MAO coating presents many irregular holes, and these holes are randomly distributed, showing the characteristics of holes in the holes (Figure 6a_3_), which is the inherent porous structure of the MAO coating.

As can be seen from Figure 6b_1_, the surface of the MAO coating becomes rough after being treated with vacuum impregnation of sodium tungstate. In the enlarged figure (Figure 6b_2_,b_3_), it can be seen that a dense and uniformly distributed honeycomb-like structure appears on the surface of the M-0.125W composite coating. This may be because in the process of loading sodium tungstate, a small amount of dissolution occurs on the surface of the MAO coating, which reacts with the aqueous solution to form Mg(OH)_2_. This is consistent with the results of the literature [46] and XPS analysis. On the one hand, the formation of the honeycomb-like structure reduces the size and number of micropores in the MAO coating to a certain extent, enhances the density of the loose layer outside the MAO coating, and then enhances the shielding ability of the coating against corrosive media. On the other hand, it provides storage space for sodium tungstate so that sodium tungstate is uniformly loaded on the surface of the MAO coating, which can be confirmed by Figure 7b. In Figure 7b, in addition to Mg, Al, O, Si, and F in the MAO coating, the W element is also detected on the surface of the M-0.125W composite coating, and the W element is uniformly distributed, which proves that sodium tungstate is uniformly loaded on the surface of the MAO coating.

It can be seen from Figure 6c_1_–c_3_ that after silanization treatment, the micropores on the surface of the MAO coating are not improved; instead, they show a tendency to expand. By comparing Figure 7a,c, it can be observed that the element C appears on the surface of the M-SG composite coating. The distribution of the element C is uniform, indicating that although silanization treatment alone cannot seal the holes of the MAO coating, an extremely thin silane film is still formed on the surface of the MAO coating.

Figure 6d_1_–g_3_, respectively, show the surface morphology of the MAO coating impregnated in sodium tungstate aqueous solutions with different concentrations and after silanization treatment. It can be seen from Figure 6d_1_–g_3_ that when the concentration of sodium tungstate is 0.025 mol/L, the micropores on the surface of the M-0.025W-SG composite coating are reduced and the honeycomb-like structure shows signs of being covered by colloidal substances (Figure 6d_2_,d_3_). When the concentration of sodium tungstate is 0.05 mol/L, the number of micropores on the surface of the M-0.05W-SG composite coating is further reduced, the gel is more obvious, and the coverage degree of the honeycomb-like structure is greater (Figure 6e_2_,e_3_). When the concentration of sodium tungstate is further increased to 0.1 mol/L or above, the surfaces of the M-0.1W-SG and M-0.125W-SG composite coatings are almost covered by colloidal substances, and the micropores and honeycomb-like structures almost disappear (Figure 6f_2_,g_2_). This shows that the loading content of sodium tungstate and the silanization treatment have a synergistic effect. With the increase in sodium tungstate concentration, the silane film is also increased, and the sealing effect on the MAO coating is more obvious. When the concentration of sodium tungstate is as high as 0.1 mol/L, the MAO coating can be completely sealed. This is consistent with the results in Figure 7d–g. In Figure 7d–g, with the increase in impregnation concentration of sodium tungstate, the content of the W element in the M-0.025W-SG, M-0.05W-SG, M-0.1W-SG, and M-0.125W-SG composite coatings increases, the content of Si and C elements also increased overall, and the sealing effect of the MAO coating becomes more obvious. The distribution of the W, Si, and C elements is uniform, indicating that the surface of the MAO coating is uniformly covered with sodium tungstate and silane film.

### 3.5. Morphology and Element Composition of Cross Section

**Figure 8:**
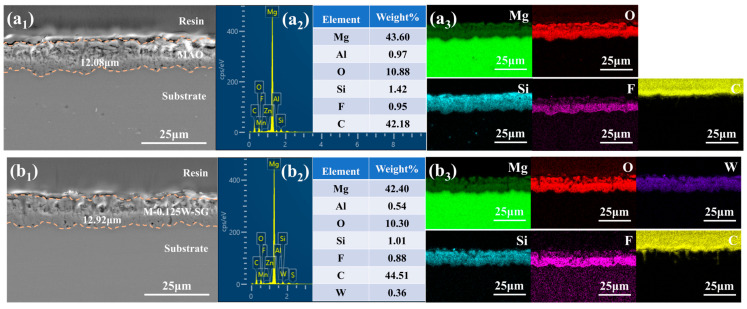
Cross-section morphology and element composition of MAO coating (**a_1_**–**a_3_**) and M-0.125W-SG composite coating (**b_1_**–**b_3_**).

Figure 8 shows the cross-section morphology, element composition, and distribution of the MAO coating and M-0.125W-SG composite coating. It can be seen from Figure 8a_1_,b_1_ that both the MAO coating and M-0.125W-SG composite coating are composed of an inner dense layer and an outer loose layer and are well combined with the magnesium alloy matrix. There are no obvious cracks at the coating/substrate interface, and the coating/substrate interface is jagged, which is attributed to the dissolution of the matrix during the coating formation process in the early stage of microarc oxidation. The thickness of the MAO coating is about 12 μm, but the thickness of M-0.125W-SG composite coating is increased to about 12.9 μm after loading sodium tungstate and silanization treatment. Compared with the MAO coating, the number of pores in the outer loose layer of the M-0.125W-SG composite coating is reduced and the pore size is decreased, which indicates that the thickness and compactness of the MAO coating of magnesium alloy are increased by loading sodium tungstate and silanization treatment.

As can be seen from Figure 8a_2_, the MAO coating is mainly composed of Mg, O, Si, and F elements. Among them, the Mg, O, and Si elements are evenly distributed in the coating, while the F element is mainly concentrated inside the coating. Additionally, after loading sodium tungstate and performing silanization treatment on the surface of the MAO coating, the W element also appears in the M-0.125W-SG composite coating. Combined with XRD and XPS, this indicates that the coating contains sodium tungstate. However, due to the coincidence of the characteristic peaks of the W element and the Si element (Figure 8b_3_), the distribution of the W element is also consistent with that of the Si element. Moreover, the C element in the two coatings mainly comes from the resin used for mounting the sample. In the outermost layer of the M-0.125W-SG composite coating, no obvious Si- and C-rich phenomena are observed, indicating that the silane film on the surface of the coating is very thin.

### 3.6. Corrosion Resistance

**Figure 9:**
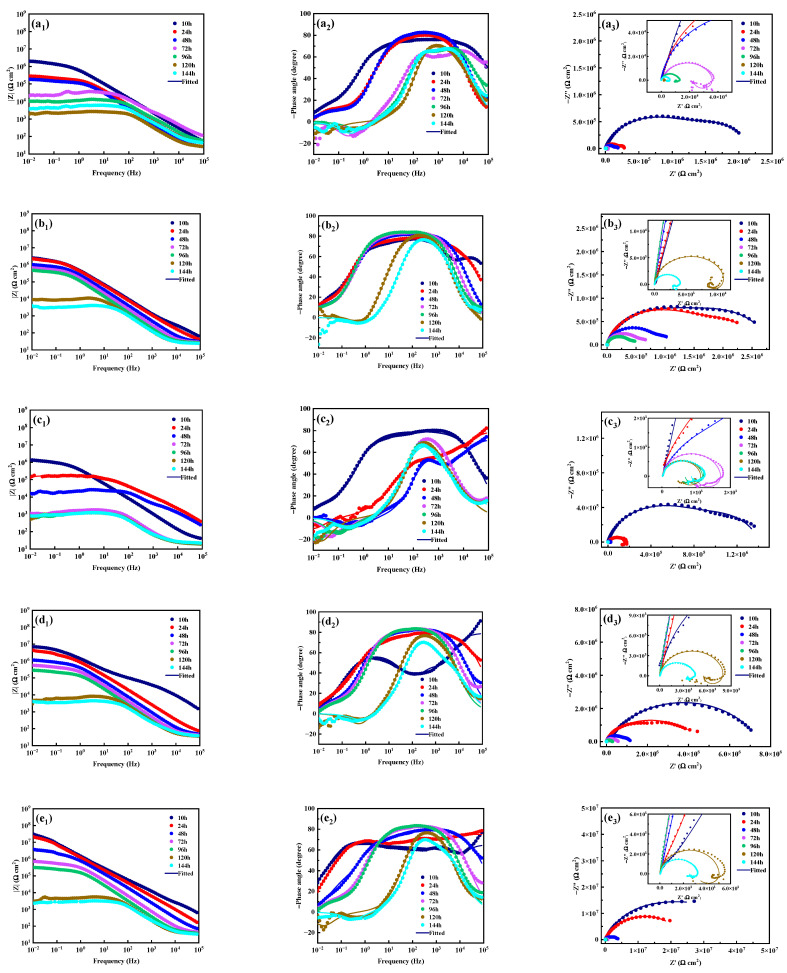

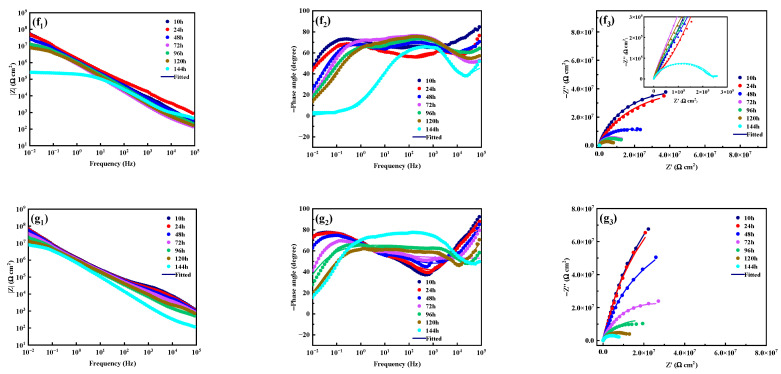
Experimental and fitting (**a_1_**–**g_1_**) modulus plot, (**a_2_**–**g_2_**) phase angle plot, and (**a_3_**–**g_3_**) Nyquist plot of MAO (**a_1_**–**a_3_**) and its composite coatings formed on AZ31B magnesium alloys: (**b_1_**–**b_3_**) M-0.125W; (**c_1_**–**c_3_**) M-SG; (**d_1_**–**d_3_**) M-0.025W-SG; (**e_1_**–**e_3_**) M-0.05W-SG; (**f_1_**–**f_3_**) M-0.1W-SG; (**g_1_**–**g_3_**) M-0.125W-SG.

**Figure 10:**
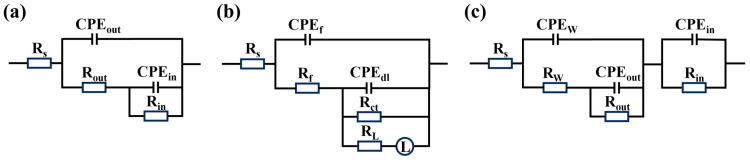
Equivalent circuit for fitting EIS of MAO and its composite coatings: (**a**) fitting EIS having two time constants; (**b**) fitting EIS having an induced reactance arc; (**c**) fitting EIS having three time constants.

Figure 9 shows the electrochemical impedance spectra and corresponding fitting results of the MAO coating and its composite coatings immersed in 3.5 wt.% NaCl solution for different times. Figure 10 shows the equivalent circuit used to fit the electrochemical impedance spectra, and the fitting results are listed in Table 3.

It can be seen from Figure 9a_2_ that within 72 h of soaking in 3.5 wt.% NaCl solution, two “peaks and valleys” appear in the phase angle curves of the MAO coating, that is, two time constants are included. At the same time, the phase angle is negative, and there are two capacitive arcs in the corresponding Nyquist diagram, which appear in the low-frequency and high-frequency regions, respectively (Figure 9a_3_). Therefore, the electrochemical impedance spectrum of the MAO coating during this stage can be fitted using the equivalent circuit shown in Figure 10a, where R_s_ represents the solution resistance, the resistance element R_out_ and its parallel constant phase angle element CPE_out_ represent the outside loose layer of the MAO coating, and the resistance element R_in_ and its parallel constant phase angle element CPE_in_ represent the inside dense layer of the MAO coating. It can be seen from Table 3 that the R_in_ value is about one order of magnitude higher than the R_out_ value when soaking for 10 h, indicating that the inside dense layer of the MAO coating plays a crucial role in resisting the penetration of the corrosive medium into the coating at the initial stage of soaking. In addition, with the extension of soaking time, the R_in_ value gradually decreases, indicating that the corrosion resistance of the coating gradually declines. When immersed for 72 h, the phase angle in the low-frequency region turns positive, and the corresponding Nyquist diagram exhibits a low-frequency induced reactance arc. This is related to the degradation absorption of metastable Mg⁺ and hydrogen release during the immersion process [47], indicating that the magnesium alloy matrix undergoes local corrosion. Consequently, the MAO coating cannot provide long-term protection for the magnesium alloy substrate. The electrochemical impedance spectrum obtained at this stage needs to be analyzed using the equivalent circuit shown in Figure 10b, where R_f_ represents the coating resistance and is connected in parallel with the constant phase angle element CPE_f_; R_ct_ stands for the charge transfer resistor, parallel to the double-layer capacitor CPE_dl_; and R_L_ is the resistance corresponding to pitting and is connected in series to the inductor L. It is worth noting that with the extension of soaking time, the corrosion resistance of the MAO coating decreases first and then increases, which is related to the formation of corrosion products [48].

When sodium tungstate is loaded into the micropores and on the surface of the MAO coating, the obtained M-0.125W composite coating is soaked for 10 h, and the phase angle curve shows three time constants (Figure 9b_2_) [49]. Therefore, the equivalent circuit shown in Figure 10c is used to fit the electrochemical impedance spectrum of the M-0.125W composite coating at 10 h, where the resistance element R_w_ and the constant phase angle element CPE_w_ represent the sodium tungstate layer. After soaking for 24 h to 96 h, the time constant becomes two, indicating that the sodium tungstate layer is lost, which is attributed to the sodium tungstate layer not being sealed and therefore dissolving in the corrosive medium. After soaking for 120 h, a low-frequency induced reactance arc appears in the Nyquist diagram, indicating that the coating is destroyed and the matrix is pitted. Compared with the MAO coating, the service life of the M-0.125W composite coating is significantly extended, indicating that loading sodium tungstate is conducive to improving the corrosion resistance of the MAO coating.

The M-SG composite coating obtained by silanization treatment on the surface of the MAO coating has the same time constant as that of the MAO coating when soaked for 10 h. However, after soaking for 24 h, the M-SG composite coating has already been destroyed and the matrix has been pitted, indicating that the corrosion resistance of the MAO coating is reduced after only silanization treatment.

The M-nW-SG composite coatings, which are obtained by vacuum-impregnating sodium tungstate of different concentrations into the pores and on the surface of the MAO coating and then performing silane sealing treatment, can be interpreted using the three-time-constant equivalent circuit shown in Figure 10c during a certain period of soaking time [50]. The resistance element R_w_ and the constant phase angle element CPE_w_ represent the sodium tungstate layer closed by the silane film. For the M-0.025W-SG composite coating, the three-time-constant response can only be maintained for 10 h. However, compared with the M-0.125W and M-SG composite coatings, the low-frequency impedance modulus value of the M-0.025W-SG composite coating is significantly increased, and the radius of the capacitive arc is remarkably enlarged. The research shows that the higher the low-frequency impedance modulus value, the larger the radius of the capacitive arc and the better the corrosion resistance of the coating [41]. This indicates that the combined treatment of sodium tungstate loading and silanization improves the corrosion resistance of the MAO coating. Meanwhile, with the increase in sodium tungstate concentration, the three-time-constant response time of the M-nW-SG composite coating lengthens. Among them, the three-time-constant response times of M-0.5W-SG and M-0.1W-SG composite coatings are extended to 24 h and 120 h, respectively, while the whole process of the M-0.125W-SG composite coating is controlled by the three-time-constant response, indicating that the M-0.125W-SG composite coating still has three layers of structure after soaking for 144 h, and its structure is complete. Furthermore, as the concentration of sodium tungstate goes up, there is a corresponding increase in both the low-frequency impedance modulus value and the radius of the capacitive arc. This, in turn, leads to a step-by-step enhancement of the overall corrosion resistance ability of the coating. When the concentration of sodium tungstate is as high as 0.125 mol/L, there is no pitting phenomenon for the M-0.125W-SG composite coating during the entire soaking process, and the R_w_ values of the M-0.125W-SG composite coating are separately 1.21 × 10^3^ and 192.10 Ω·cm^2^ after immersing for 120 h and 144 h; at the same time, the radius of the capacitive arc is the largest and the corrosion resistance of the coating is the strongest.

**Figure 11:**
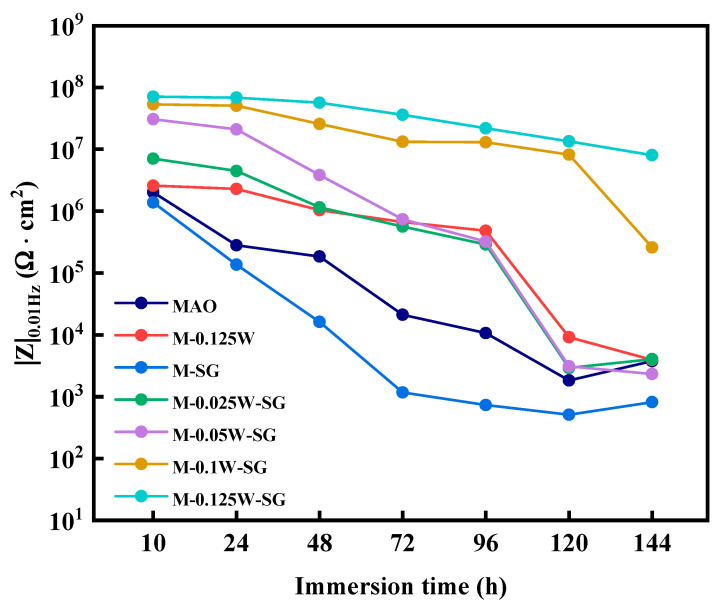
Low-frequency impedance modulus values of MAO and its composite coatings immersed in 3.5 wt.% NaCl solution for different times.

Figure 11 shows the low-frequency impedance modulus values (|Z|_0.01 Hz_) of different coatings soaked in 3.5 wt.% NaCl solution for different periods of time. Studies have shown that the larger the low-frequency impedance modulus, the better the overall corrosion resistance of the coating [51]. During the entire soaking process, the overall modulus values are in the following order: M-0.125W-SG > M-0.1W-SG > M-0.05W-SG > M-0.025W-SG > M-0.125W > MAO > M-SG. This indicates that only silanization treatment on the surface of the MAO coating reduces the corrosion resistance of the MAO coating. However, when the surface of the MAO coating is loaded with sodium tungstate and undergoes silanization treatment, the corrosion resistance of the coating is increased. The higher the concentration of sodium tungstate, the stronger the corrosion resistance of the coating. The M-0.125W-SG composite coating has the strongest corrosion resistance.

In addition, with the extension of soaking time, the |Z|_0.01Hz_ of the coating decreases on the whole, indicating that the corrosion resistance of the coating decreases gradually. The |Z|_0.01Hz_ of the MAO coating shows a linear decline during the whole soaking period. When soaked for 72 h, the |Z|_0.01Hz_ is 2.11 × 10^4^ Ω·cm^2^, which is similar to that of the magnesium alloy substrate [52], indicating that the coating has lost its protective ability for the magnesium alloy matrix. Yet at 144 h, the |Z|_0.01Hz_ of the MAO coating increases. This is attributed to the gradual accumulation of corrosion products on the surface of the magnesium alloy matrix, which hinders the transfer process of the etching medium. Compared with that of the MAO coating, the reduction extent of |Z|_0.01Hz_ of the M-0.125W composite coating decreases significantly from 10 h to 72 h. However, the |Z|_0.01Hz_ of the M-0.125W composite coating decreases sharply after 96 h and the coating fails at 120 h; however, the failure time of the M-0.125W composite coating is delayed by 48 h compared to the MAO coating, indicating an extended service life. After the MAO coating undergoes silanization treatment alone, the obtained M-SG composite coating has a greater decrease in |Z|_0.01Hz_. Moreover, it is lower than that of the MAO coating throughout the soaking process, demonstrating worse corrosion resistance than the MAO coating. For the M-nW-SG composite coating that is loaded with sodium tungstate and treated by silanization, the decreased extent of the |Z|_0.01Hz_ at 10–72 h further lessens with the increase in sodium tungstate concentration. However, the |Z|_0.01Hz_ of M-0.025W-SG and M-0.05W-SG composite coatings still decreases sharply after 96 h and fails at 120 h, which is attributed to the low loading content of sodium tungstate and the poor sealing effect of the silane film. However, the sudden drop in the |Z|_0.01Hz_ of the M-0.1W-SG composite coating takes place only after 120 h, and the |Z|_0.01Hz_ reaches 2.59 × 10^5^ Ω·cm^2^ when soaked for 144 h. Moreover, the M-0.125W-SG composite coating has excellent stability during the whole soaking process due to the greater amount of sodium tungstate and good closure effect of the silane film. After 144 h soaking, the |Z| _0.01Hz_ of the M-0.125W-SG composite coating is still maintained at 8.06 × 10^6^ Ω·cm^2^, which is higher than that of the initial stage of the MAO coating (10 h), showing the best long-term corrosion resistance.

### 3.7. Corrosion Morphology

**Figure 12:**
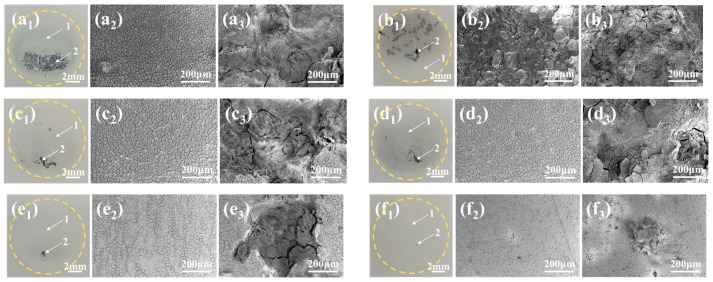
Macroscopic morphology (**a_1_**–**f_1_**) and microscopic morphology of slightly corroded area (**a_2_**–**f_2_**) and severely corroded area (**a_3_**–**f_3_**) of different coatings after immersion in 3.5 wt.% NaCl solution for 168 h: MAO (**a_1_**–**a_3_**), M-SG (**b_1_**–**b_3_**), M-0.025W-SG (**c_1_**–**c_3_**), M-0.05W-SG (**d_1_**–**d_3_**), M-0.1W-SG (**e_1_**–**e_3_**), and M-0.125W-SG (**f_1_**–**f_3_**). Dashed circles in (**a_1_**–**f_1_**) show the exposed area (1 cm^2^), 1 represents a slightly corroded area, and 2 indicates a severely corroded area.

Figure 12 shows the macro- and micromorphologies of MAO and its composite coatings after soaking in 3.5 wt.% NaCl solution for 168 h. As can be seen from the macroscopic morphology of Figure 12a_1_–f_1_, different sizes of corrosion pits appear on the macroscopic surface of MAO and its composite coatings after soaking for 168 h. Among them, the area of the corrosion pit of the MAO coating and the M-SG composite coating is relatively large, indicating that these two coatings are more severely corroded. After only the loading of sodium tungstate on the surface of the MAO coating, the corrosion pit area of the obtained M-0.125W composite coating decreases to some extent. When the MAO coating with sodium tungstate is further treated by silanization, the corrosion pit area of the synthesized M-nW-SG composite coating decreases even more. Moreover, the area of the corrosion pit on the surface of the M-nW-SG composite coating decreases as the concentration of sodium tungstate increases. When the concentration of sodium tungstate is 0.125 mol/L, almost no corrosion pits appear on the surface of the prepared M-0.125W-SG composite coating (as shown in Figure 12f_1_), indicating that its resistance to pitting corrosion is the strongest. This can be attributed to the high loading content of sodium tungstate and the dense structure of the coating. It also demonstrates that loading sodium tungstate and undergoing silanization treatment have a synergistic effect on enhancing the corrosion resistance of the magnesium alloy with MAO coating.

Figure 12a_2_–f_2_,a_3_–f_3_, respectively, display the micromorphologies of the slightly corroded area and severely corroded area on the area of the coating exposed to 3.5 wt.% NaCl solution, corresponding to region 1 and region 2 of Figure 12a_1_–f_1_. As can be observed from Figure 12a_2_–f_2_,a_3_–f_3_, although there is no spalling of the coating in region 1, the surface of the coating in this region is covered with grid-like corrosion cracks. This may be caused by the infiltration of the corrosive medium into the coating, resulting in the generation of internal stress. On the surface of the MAO coating, the corrosion cracks in region 1 are more densely distributed. In region 2, the corrosion pits are deeper and larger, with almost no coating residue remaining (as shown in Figure 12a_3_). After sodium tungstate is loaded on the surface of the MAO coating and silanization treatment is performed, the mesh-like corrosion cracks in region 1 and the size of the corrosion pits in region 2 gradually improve as the concentration of sodium tungstate increases. When the concentration of the sodium tungstate is 0.125 mol/L, there are almost no obvious mesh-like corrosion cracks in region 1. In addition, only a small amount of corrosion products accumulate on the surface of region 2, indicating that the corrosion resistance of M-nW-SG composite coatings gradually increases with the increase in the concentration of sodium tungstate, which is attributed to the greater amount of sodium tungstate in the coating and the enhancement of the silanization sealing effect (Figure 6d_1_–g_3_).

### 3.8. Scratch Test

**Figure 13:**
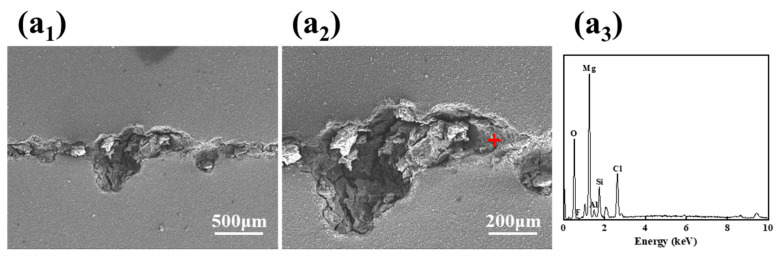

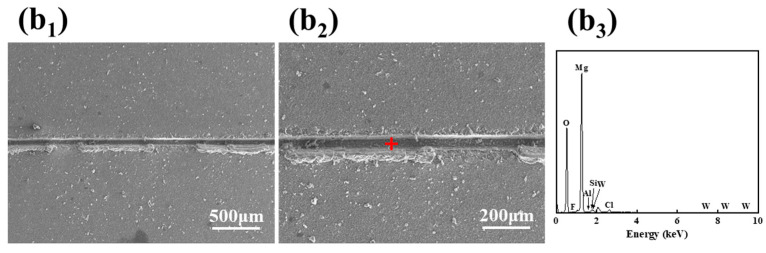
Artificial scratch experiment on (**a_1_**–**a_3_**) MAO coating and (**b_1_**–**b_3_**) M-0.125W-SG composite coating after immersion in 3.5 wt.% NaCl solution for 96 h. (**a_1_**,**b_1_**) SEM micrographs; (**a_2_**,**b_2_**) enlarged SEM micrographs; (**a_3_**,**b_3_**) EDS spectra corresponding to the “Red +” regions shown in Figure 13 (**a_2_**,**b_2_**), among which the “Red +”is the representative EDS test point.

In order to further evaluate the corrosion resistance of the coating, artificial long scratch defects are prepared on the MAO coating and M-0.125W-SG composite coating by using a sharp knife to expose the magnesium alloy substrate, and then they are soaked in 3.5 wt.% NaCl solution for 96 h; their corrosion morphology is shown in Figure 13. As can be seen from Figure 13, after the MAO coating is soaked for 96 h, the corrosive medium erodes along the scratch zone to the surrounding area and inside the matrix, respectively, resulting in serious corrosion of the magnesium alloy matrix and MAO coating, and a large number of corrosion products accumulate in the corrosion pit. The results show that not only can the MAO coating play a protective role in the undamaged state but also, when the magnesium alloy matrix is exposed in the corrosive medium, it cannot respond to the corrosion behavior of the damaged area.

For the M-0.125W-SG composite coating, except for the defects caused by artificial scratches, no new corrosion pits are observed, which indicates good corrosion resistance to the magnesium alloy substrate. This may be attributed to the release of sodium tungstate in the micropores and on the surface of the MAO coating. And the released sodium tungstate adsorbs on the surface of the magnesium alloy substrate, shields the matrix, and inhibits the corrosion process, thereby enhancing the protective effect on the magnesium alloy substrate.

## 4. Discussion

**Figure 14:**
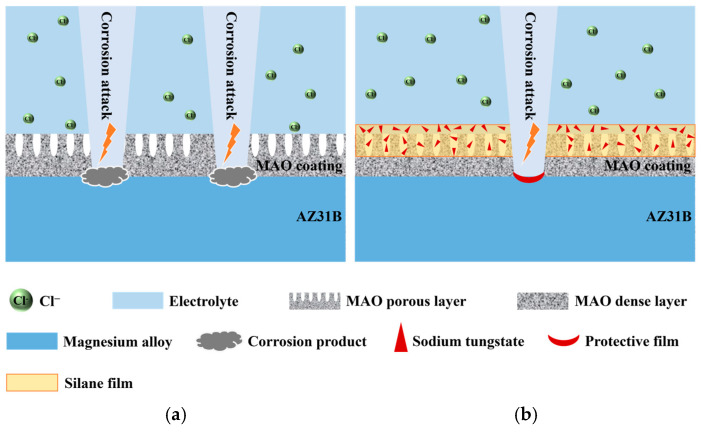
Schematic illustration of corrosion process of (**a**) MAO coating and (**b**) M-nW-SG composite coating in 3.5 wt.% NaCl solution.

Microarc oxidation is a process of continuous breakdown, reaction, condensation, and deposition. The obtained MAO coating blocks the direct contact between the corrosive medium and the magnesium alloy matrix, thus playing a role in corrosion protection for the magnesium alloy matrix. However, in this process, there are inevitably micropores and microcracks and other defects in the MAO coating. The corrosive medium can penetrate the coating through these defects and reach the magnesium alloy matrix, thereby corroding the matrix. Therefore, the micropores and microcracks on the surface of the MAO coating are the main factors limiting the long-term corrosion resistance of the MAO coating. In this study, as can be observed from Figure 9a_3_, an induced reactance arc appears when the MAO coating is soaked in 3.5 wt.% NaCl solution for 72 h. This indicates that the corrosive medium has penetrated to the surface of the magnesium alloy matrix, resulting in corrosion of the magnesium alloy matrix. As seen in Figure 12a_2_, after soaking for 168 h, a large number of grid-like corrosion cracks occur in the slightly corroded area on the surface of the MAO coating. This can be attributed to the fact that Cl⁻ ions and H_2_O in the NaCl solution penetrate into the interface between the coating and the substrate through the microdefects of the MAO coating. Electrochemical reactions then take place (as indicated by Equations (9)–(11)), generating corrosion products in the microdefects of the coating. As a result, a stress concentration area is formed. When the local stress level exceeds the strength limit of the coating, cracks will occur along the fragile area of the coating. These cracks will continue to expand under the combined action of tensile stress and corrosive medium, and gradually form macroscopic cracks. When the corrosion is more severe, it will cause the coating to peel off, resulting in corrosion pits, as shown in Figure 12a_3_, and the schematic illustration of corrosion process of MAO coating is dispalyed in Figure 14a.

MgO + H_2_O + Cl^−^ → Mg(OH)_2_ + Cl^−^ (9)

Mg + 2H_2_O → Mg(OH)_2_ + H_2_ (10)

Mg(OH)_2_ + 2Cl^−^ → MgCl_2_ + 2OH^−^ (11)

When sodium tungstate is loaded on the surface of the MAO coating by vacuum impregnation, the obtained M-0.125W composite coating presents a honeycomb-like structure on its surface. This structure reduces the size and number of micropores in the MAO coating to a certain extent, enhances the compactness of the outer loose layer of the MAO coating, and further improves the shielding ability of the MAO coating against corrosive media. Furthermore, the loaded sodium tungstate can combine with Mg^2^⁺ to form an insoluble magnesium tungstate protective film (as shown in Equation (12)). This effectively prevents the erosion of Cl^−^ ions and H_2_O. As a result, after soaking for 10 h, the R_out_ value of the M-0.125W composite coating increases from 9.4 × 10⁵ Ω·cm^2^ to 1.36 × 10⁶ Ω·cm^2^ compared with the MAO coating, and it begins to fail after 120 h. At the same time, after soaking for 144 h, the area of corrosion pits on the surface of the M-0.125W composite coating is also greatly reduced.

Mg^2+^ + WO_4_^2−^ → MgWO_4_ (12)

However, when the MAO coating is only subjected to silanization treatment, the formation of a silane film thick enough to effectively close the micropores of the MAO coating is difficult. This is because only a small number of hydroxyl groups on the surface of the MAO coating can condense with the pre-hydrolyzed silane to form M-O-Si bonds (where M represents metal atoms). In addition, the MAO coating is mainly composed of MgO, Mg_2_SiO_4_, and MgF_2_. Since MgO is an alkaline oxide, which can react with the acid silane pre-hydrolysate to dissolve the coating, it can thus result in an increase in the micropore size of the MAO coating [53]. However, micropores are the channels through which the corrosive medium penetrates into the coating and corrodes the magnesium alloy substrate, and so the increase in the micropores will inevitably lead to the decrease in the corrosion resistance of the coating. Therefore, compared with MAO coating, the corrosion resistance of M-SG composite coating obtained only by silanization treatment is reduced.

For the M-nW-SG composite coating, which is obtained by vacuum impregnation of sodium tungstate and silanization treatment on the surface of the MAO coating, apart from the protective mechanism of the M-0.125W composite coating, the sealing effect of silanization on the MAO coating with sodium tungstate is even more crucial. When sodium tungstate is filled by vacuum impregnation on the surface of the MAO coating, a small amount of dissolution takes place on the surface of the MAO coating and Mg(OH)_2_ is formed, which can provide some hydroxyl groups. The pre-hydrolyzed product of silane combines with the hydroxyl group via the Si-OH bond to form an M-O-Si bond (where M represents the metal atom), thereby realizing the sealing effect on the MAO coating with sodium tungstate and enhancing the shielding ability of the coating against corrosive media. In addition, the silane film itself not only possesses hydrophobic properties but also serves as a physical barrier, preventing the corrosive medium from propagating to the interior of the MAO coating and hindering the diffusion and dissolution of sodium tungstate into the corrosion solution. The possible mechanism is presented in Figure 14b.

As the concentration of sodium tungstate increases, the content of the W element in the coating grows and the sealing effect of the silanization treatment becomes better; the M-nW-SG composite coatings are also more compact, presenting a synergistic effect. Therefore, with the rising concentration of sodium tungstate, the corrosion resistance of the coating is enhanced. Among the coatings tested, the M-0.125W-SG composite coating has the best corrosion resistance. When soaked in 3.5 wt.% NaCl solution for 144 h, its impedance modulus value can still remain at 8.06 × 10⁶ Ω·cm^2^, which is approximately three orders of magnitude higher than that of the MAO coating.

Obviously, loading sodium tungstate and performing silanization treatment on the surface of the MAO coating can not only keep the sodium tungstate directly loaded on the surface of the MAO coating from entering the corrosive medium to cause waste, thereby improving the utilization efficiency of sodium tungstate, but also enhance the sealing effect of the silanization treatment. Consequently, the overall corrosion resistance of the M-nW-SG composite film is improved.

## 5. Conclusions

1. Loading sodium tungstate and undergoing silane treatment displays a synergistic effect on an MAO coating formed on magnesium alloy. When only sodium tungstate is loaded onto the surface of the magnesium alloy MAO coating, a passivation film can be generated through the reaction between sodium tungstate and Mg^2^⁺ ions. This film can delay the penetration of corrosive media into the coating to corrode the substrate and further improve the corrosion resistance of the MAO coating. Nevertheless, when only the surface of the MAO coating is silanized, the size of the micropores within the coating increases due to the reaction of the silane hydrolysate with the substances in the coating. As a result, the corrosion resistance of the coating is decreased. When sodium tungstate is loaded onto the surface of the MAO coating and subsequently silanized, not only can a protective film be formed as a result of the reaction between sodium tungstate and Mg^2^⁺ ions but also the silanization treatment is capable of sealing the MAO coating. In this way, it significantly boosts the corrosion resistance of the magnesium alloy MAO coating.
2. The concentration of sodium tungstate also has a significant effect on magnesium alloy MAO composite coating. With the increase in the impregnation concentration of sodium tungstate, the loaded content of sodium tungstate in the M-nW-SG composite coating increases and the sealing effect of the silanization treatment is better. When the impregnation concentration of sodium tungstate is 0.1 mol/L or above, the MAO coating with sodium tungstate can be completely sealed. Among the samples tested, when the M-0.125W-SG composite coating was soaked in 3.5 wt.% NaCl solution for 144 h, its impedance modulus remained at 8.06 × 10^6^ Ω·cm^2^, which is about three orders of magnitude higher than that of the MAO coating, and it had the best corrosion resistance. This provides a reference for the improvement of corrosion resistance of MAO and its composite coatings.

## Figures and Tables

**Table 1:** Chemical composition of AZ31B magnesium alloy (wt.%).

Al	Zn	Mn	Si	Cu	Ni	Fe	Mg
3.1%	0.8%	0.15%	0.06%	0.03%	0.02%	0.01%	Balance

**Table 2:** Abbreviation of MAO coating and its composite coatings.

Ab. Name	Na_2_WO_4_ g/L	GPTMS
MAO	−	−
M-0.125W	0.125	−
M-SG	−	+
M-0.025W-SG	0.025	+
M-0.05W-SG	0.05	+
M-0.1W-SG	0.1	+
M-0.125W-SG	0.125	+

**Table 3:** Fitted EIS data of MAO and its composite coatings in 3.5 wt.% NaCl corrosive medium during 144 h.

Sample	Immersion Time (h)	R_W_ (Ω·cm^2^)	CPE_W_	*n*	R_out_ (Ω·cm^2^)	CPE_out_	*n*	R_in_ (Ω·cm^2^)	CPE_in_	*n*	R_L_	L
			Y_0_ (nΩ^−1^ cm^−2^ s^n^)			Y_0_ (nΩ^−1^ cm^−2^ s^n^)			Y_0_ (nΩ^−1^ cm^−2^ s^n^)			
MAO	10 h				9.40 × 10^5^	3.72 × 10^−6^	0.85	1.22 × 10^6^	2.99 × 10^−7^	0.85		
	24 h				1.11 × 10^5^	1.80 × 10^−5^	0.86	1.74 × 10^5^	5.08 × 10^−7^	0.91		
	48 h				1.25 × 10^5^	6.45 × 10^−7^	0.93	7.17 × 10^4^	3.14 × 10^−5^	0.71		
	72				6.96 × 10^3^	2.79 × 10^−7^	0.81	3.00 × 10^4^	4.47 × 10^−8^	1	3.35 × 10^4^	2.97 × 10^4^
	96 h				4.12 × 10^3^	4.22 × 10^−7^	0.87	8.92 × 10^3^	6.93 × 10^−8^	1	1.64 × 10^4^	5.01 × 10^3^
	120 h				155.50	5.05 × 10^−7^	0.96	2.33 × 10^3^	2.96 × 10^−7^	0.95	5.01 × 10^3^	3.41 × 10^4^
	144 h				1.74 × 10^3^	4.92 × 10^−7^	0.91	4.31 × 10^3^	1.59 × 10^−7^	1	5.36 × 10^3^	4.14 × 10^3^
M-0.125W	10 h	137.70	1.18 × 10^−7^	0.97	1.36 × 10^6^	3.42 × 10^−6^	0.88	1.43 × 10^6^	4.58 × 10^−8^	0.86		
	24 h				1.37 × 10^6^	4.94 × 10^−7^	0.90	1.29 × 10^6^	3.81 × 10^−6^	0.76		
	48 h				7.38 × 10^5^	6.91 × 10^−7^	0.92	3.65 × 10^5^	1.41 × 10^−5^	0.90		
	72				2.28 × 10^5^	2.25 × 10^−5^	0.92	4.74 × 10^5^	1.04 × 10^−6^	0.94		
	96 h				3.46 × 10^5^	1.52 × 10^−6^	0.95	1.64 × 10^5^	3.22 × 10^−5^	0.90		
	120 h				7.76 × 10^3^	1.89 × 10^−6^	0.96	4.26 × 10^3^	1.25 × 10^−5^	0.44	1.02 × 10^3^	7.40 × 10^2^
	144 h				2.91 × 10^3^	2.14 × 10^−6^	0.94	1.24 × 10^3^	1.28 × 10^−7^	1	4.23 × 10^2^	2.61 × 10^2^
M-SG	10 h				8.40 × 10^5^	4.57 × 10^−7^	0.88	5.60 × 10^5^	5.52 × 10^−6^	0.88		
	24 h				6.45 × 10^3^	1.87 × 10^−8^	0.90	1.67 × 10^5^	2.36 × 10^−7^	0.67	3.73 × 10^5^	6.72 × 10^6^
	48 h				4.76 × 10^3^	8.35 × 10^−8^	0.82	2.01 × 10^4^	6.05 × 10^−8^	0.96	4.07 × 10^4^	1.41 × 10^4^
	72				12.15	3.27 × 10^−7^	0.97	1.69 × 10^3^	2.59 × 10^−6^	0.94	2.23 × 10^3^	5.794 × 10^3^
	96 h				32.55	2.28 × 10^−6^	0.94	1.14 × 10^3^	2.28 × 10^−6^	0.94	1.44 × 10^3^	3.04 × 10^3^
	120 h				32.33	1.78 × 10^−6^	0.96	1.08 × 10^3^	2.01 × 10^−6^	0.97	830.20	1.07 × 10^4^
	144 h				11.9	8.68 × 10^−7^	0.92	1.23 × 10^3^	5.29 × 10^−6^	0.90	2.60 × 10^3^	1.71 × 10^3^
M-0.025W-SG	10 h	1.87 × 10^4^	1.29 × 10^−9^	1	1.08 × 10^5^	5.98 × 10^−8^	0.68	7.46 × 10^6^	1.22 × 10^−7^	0.73		
	24 h				1.26 × 10^6^	1.75 × 10^−7^	0.88	3.52 × 10^6^	3.67 × 10^−7^	0.51		
	48 h				7.19 × 10^5^	3.52 × 10^−7^	0.92	4.60 × 10^5^	4.07 × 10^−6^	0.70		
	72				3.94 × 10^5^	5.91 × 10^−7^	0.93	1.66 × 10^5^	1.34 × 10^−5^	0.84		
	96 h				1.97 × 10^5^	9.05 × 10^−7^	0.94	8.67 × 10^4^	2.53 × 10^−5^	0.86		
	120 h				23.95	2.98 × 10^−7^	0.96	8.12 × 10^3^	7.72 × 10^−7^	0.92	1.68 × 10^4^	5.69 × 10^3^
	144 h				249.80	1.24 × 10^−6^	0.89	4.23 × 10^3^	3.10 × 10^−7^	1	1.43 × 10^4^	3.90 × 10^3^
M-0.05W-SG	10 h	464.10	1.84 × 10^−8^	1	3.99 × 10^7^	2.20 × 10^−7^	0.69	9.22 × 10^6^	5.49 × 10^−7^	1		
	24 h	4.50 × 10^4^	5.00 × 10^−7^	0.81	6.21 × 10^3^	7.36 × 10^−8^	0.88	2.46 × 10^7^	1.09 × 10^−7^	0.75		
	48 h				4.06 × 10^5^	1.71 × 10^−7^	0.89	3.71 × 10^6^	2.37 × 10^−7^	0.47		
	72				4.76 × 10^5^	4.08 × 10^−7^	0.92	2.68 × 10^5^	7.35 × 10^−6^	0.76		
	96 h				2.22 × 10^5^	7.45 × 10^−7^	0.94	9.60 × 10^4^	2.33 × 10^−5^	0.85		
	120 h				19.50	2.76 × 10^−7^	0.98	4.99 × 10^3^	7.43 × 10^−7^	0.96		
	144 h				324.30	9.98 × 10^−7^	0.93	2.86 × 10^3^	3.14 × 10^−7^	1	9.24 × 10^3^	4.97 × 10^3^
M-0.1W-SG	10 h	1.54 × 10^3^	1.03 × 10^−8^	0.99	5.67 × 10^5^	7.79 × 10^−7^	0.59	8.77 × 10^7^	1.67 × 10^−7^	0.89		
	24 h	1.78 × 10^3^	2.71 × 10^−9^	1	5.08 × 10^4^	2.25 × 10^−7^	0.70	1.00 × 10^8^	1.36 × 10^−7^	0.79		
	48 h	1.07 × 10^3^	5.86 × 10^−8^	0.85	1.15 × 10^5^	4.35 × 10^−7^	0.75	3.05 × 10^7^	1.88 × 10^−7^	0.83		
	72	468.10	2.03 × 10^−7^	0.79	5.27 × 10^3^	5.47 × 10^−8^	0.92	1.5 × 10^7^	6.22 × 10^−9^	1		
	96 h	1.33 × 10^3^	6.86 × 10^−8^	0.85	6.31 × 10^5^	7.306 × 10^−8^	0.84	1.41 × 10^7^	9.78 × 10^−8^	0.66		
	120 h	346.10	1.27 × 10^−6^	0.66	2.83 × 10^5^	1.54 × 10^−7^	0.88	8.84 × 10^6^	1.35 × 10^−7^	0.58		
	144 h				1.59 × 10^3^	3.89 × 10^−7^	0.64	2.45 × 10^5^	3.52 × 10^−8^	0.94		
M-0.125W-SG	10 h	1.94 × 10^4^	2.83 × 10^−9^	0.96	2.23 × 10^6^	5.91 × 10^−7^	0.53	3.57 × 10^8^	1.92 × 10^−7^	0.94		
	24 h	1.39 × 10^4^	3.47 × 10^−9^	0.95	1.57 × 10^6^	6.04 × 10^−7^	0.54	3.13 × 10^8^	2.00 × 10^−7^	0.94		
	48 h	4.42 × 10^3^	1.84 × 10^−9^	1	1.89 × 10^6^	6.49 × 10^−7^	0.53	1.55 × 10^8^	2.23 × 10^−7^	0.94		
	72 h	2.39 × 10^3^	4.36 × 10^−7^	0.59	7.81 × 10^4^	6.47 × 10^−7^	0.78	7.89 × 10^7^	4.12 × 10^−7^	0.99		
	96 h	421.50	5.24 × 10^−9^	1	982.3	3.54 × 10^−7^	1	3.76 × 10^7^	2.58 × 10^−7^	0.73		
	120 h	1.21 × 10^3^	3.39 × 10^−9^	1	2.38 × 10^4^	1.07 × 10^−6^	0.68	1.53 × 10^7^	2.48 × 10^−7^	0.74		
	144 h	192.10	8.04 × 10^−7^	0.7	4.72 × 10^6^	2.74 × 10^−7^	1	4.53 × 10^6^	4.28 × 10^−7^	0.85		

## Data Availability

The original contributions presented in this study are included in the article. Further inquiries can be directed to the corresponding authors.
